# PD34 Advances In Medical Device Evaluation Within The National Committee for Health Technology Incorporation (CONITEC)

**DOI:** 10.1017/S0266462325101682

**Published:** 2025-12-29

**Authors:** Daniele Marques, Joana Ferreira da Silva, Aíla Coelho do Carmo, Luciene Bonan

## Abstract

**Introduction:**

CONITEC plays a central role in health technology assessment (HTA), particularly in advancing the evaluation of medical devices (MDs). Tools such as horizon scanning (HS) and the Committee on Products and Procedures enhance its methodologies. This study examined the evolution of MD evaluation over time.

**Methods:**

The requests submitted to CONITEC for analysis were carefully identified, quantified, and analyzed in detail. Special attention was given to requests that were specifically directed to the evaluation and recommendation processes carried out by the Commission’s various committees. These requests were thoroughly reviewed to ensure that all relevant aspects were considered. Subsequently, the MD-related proposals were carefully examined, with a particular focus on the incorporation recommendations provided by CONITEC. In addition, the evaluations that led to the creation of HS analyses were closely analyzed, providing insights into the decision-making process and the development of these important evaluations.

**Results:**

Over the last 12 years, CONITEC received 1,082 HTA requests, with 660 originating internally and 422 externally. Of these, 832 were related to medicines, 174 to procedures, and 76 to products. Of the product and procedure proposals, 159 were recommended for incorporation, 35 were not recommended, five resulted in exclusion, one resulted in non-exclusion, and six were closed by CONITEC’s decision. Approximately 18 percent of the MD-related requests led to the creation of HS analyses, particularly in 2023 and 2024 when all demands in this area resulted in HS analyses, reflecting improvements in the evaluation process.

**Conclusions:**

The evaluation of MDs is a rapidly growing field within HTA, requiring the adoption of specific methods for progress. These methods include tools and databases focused on HS as well as tailored methodological guidelines. CONITEC has continuously developed tools to enhance decision-making on the incorporation of MDs into the Unified Health System.

